# Estimation of Antenna Pose in the Earth Frame Using Camera and IMU Data from Mobile Phones

**DOI:** 10.3390/s17040806

**Published:** 2017-04-08

**Authors:** Zhen Wang, Bingwen Jin, Weidong Geng

**Affiliations:** College of Computer Science and Technology, Zhejiang University, Hangzhou 310058, China; wangzh_cs@zju.edu.cn (Z.W.); jinbw@zju.edu.cn (B.J.)

**Keywords:** pose estimation, sensor fusion, mobile phone

## Abstract

The poses of base station antennas play an important role in cellular network optimization. Existing methods of pose estimation are based on physical measurements performed either by tower climbers or using additional sensors attached to antennas. In this paper, we present a novel non-contact method of antenna pose measurement based on multi-view images of the antenna and inertial measurement unit (IMU) data captured by a mobile phone. Given a known 3D model of the antenna, we first estimate the antenna pose relative to the phone camera from the multi-view images and then employ the corresponding IMU data to transform the pose from the camera coordinate frame into the Earth coordinate frame. To enhance the resulting accuracy, we improve existing camera-IMU calibration models by introducing additional degrees of freedom between the IMU sensors and defining a new error metric based on both the downtilt and azimuth angles, instead of a unified rotational error metric, to refine the calibration. In comparison with existing camera-IMU calibration methods, our method achieves an improvement in azimuth accuracy of approximately 1.0 degree on average while maintaining the same level of downtilt accuracy. For the pose estimation in the camera coordinate frame, we propose an automatic method of initializing the optimization solver and generating bounding constraints on the resulting pose to achieve better accuracy. With this initialization, state-of-the-art visual pose estimation methods yield satisfactory results in more than 75% of cases when plugged into our pipeline, and our solution, which takes advantage of the constraints, achieves even lower estimation errors on the downtilt and azimuth angles, both on average (0.13 and 0.3 degrees lower, respectively) and in the worst case (0.15 and 7.3 degrees lower, respectively), according to an evaluation conducted on a dataset consisting of 65 groups of data. We show that both of our enhancements contribute to the performance improvement offered by the proposed estimation pipeline, which achieves downtilt and azimuth accuracies of respectively 0.47 and 5.6 degrees on average and 1.38 and 12.0 degrees in the worst case, thereby satisfying the accuracy requirements for network optimization in the telecommunication industry.

## 1. Introduction

Antenna pose has always played an important role in cellular network planning and optimization, from the era of the 2G network [1] to the present day (e.g., [2,3]). It directly affects signal coverage, soft handover and interference between cells [4] and indirectly affects other network performance indicators, such as quality of service [5], and network configuration parameters, such as transmission power [6]. Thus, determining the pose of an antenna during installation and monitoring its subsequent changes in pose are important tasks.

The pose of an antenna is typically parameterized in terms of its downtilt (or elevation) and azimuth angles (e.g., in [4]), which are formally defined with respect to the direction of the main lobe [7]. For the time being, there are two popular approaches to measure the antenna pose in the industry. The first method is to measure the downtilt and azimuth angles manually by a person using an inclinometer and a compass; the second one is to employ specialized sensors, such as the Antenna WASP [8] from 3Z Telecom™, or portable measurement devices equipped with internal sensors, such as the antenna alignment tool (AAT) [9] from Sunlight™, to facilitate the measurement process.

However, both methods have their limitations. For the manual measurement, because numerous antennas are mounted on towers that are high off the ground and electrically powered, reaching these antennas takes much effort and poses a high risk for workers. Moreover, it is difficult to guarantee the accuracy and precision of such manual measurements because of individual differences among workers. As for the second solution with sensors, on the one hand, since a single sensor unit like the WASP costs a few tens of U.S. dollars, the gross overhead becomes enormous when the total number of antennas is considered for a large mobile network; on the other hand, portable measurement devices like AAT are usually expensive, and workers still must physically access the antennas to use them.

The recent proliferation of mobile phones with various types of built-in sensors, especially cameras and inertial measurement units (IMUs), has given rise to a wide range of interesting new applications and algorithms [10] that rely on the fusion of visual and inertial information for use in many fields: for example, object recognition [11], 3D reconstruction [12,13], tracking [13,14] and pose estimation [15]. These studies have inspired us to propose a novel non-contact solution to the measurement problem of the antenna pose in the Earth frame using the camera and IMU data from mobile phones.

**Figure 1:**
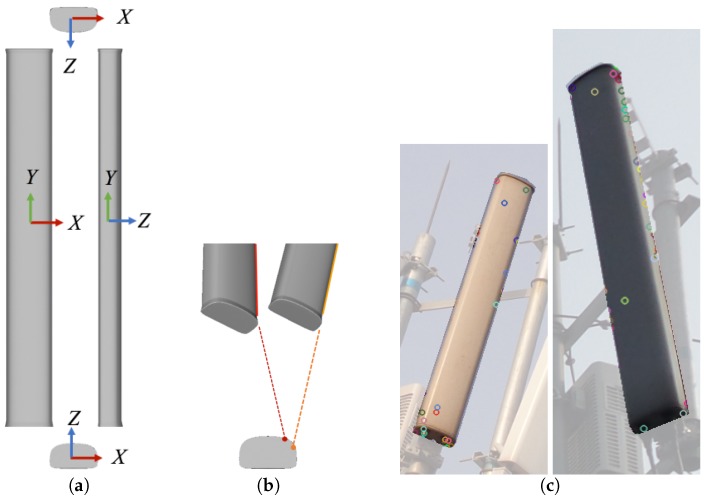
The simple geometry and textureless surface of an antenna. (**a**) Antenna geometry (front view, right view, top view and bottom view); (**b**) seemingly corresponding edges in images do not coincide when back-projected onto the 3D geometry; (**c**) a few stable SIFT features are detected on antenna surfaces (the same antenna viewed from two angles).

Two technical challenges arise when designing this non-contact approach. First, antennas are only sparsely textured and usually have simple shapes with smooth surfaces (see Figure 1a), providing a few of the distinctive features and stable matches (see Figure 1b,c) that are usually required for existing feature-based pose estimation methods. Second, the IMU sensors in mobile phones are usually ultra-low-cost (consumer-level) microelectromechanical system (MEMS) sensors with poor accuracy [16,17,18]; however, accuracy is of key importance for industry applications, such as network optimization [19].

To address these challenges, we design and develop our solution based on adequate consideration of the characteristics of antennas and the sensors in mobile phones. First, we introduce a 3D antenna model and describe the visual pose estimation problem for an antenna as a direct 2D-3D matching problem based on the outer contours of the antenna to avoid the influence from the antenna’s lack of geometric and textural features. This approach requires prior knowledge of the antenna’s 3D geometry, but this is not yet an excessive requirement because of the limited number of different antenna products that are currently in use. Second, to improve the accuracy of pose estimation, we develop a coarse-to-fine strategy for antenna pose estimation, in which we first find an approximate pose automatically by exploiting the shape characteristics of the antenna and reduce the original unconstrained candidate pose search space to a constrained one, and we then seek an optimal solution in this reduced space using global optimization techniques. Moreover, to reduce the visual-inertial fusion error of mobile phones, we also propose a new camera-IMU calibration method for accurate calculation of the relative poses between the relevant sensors.

Therefore, we are able to build up a non-contact antenna pose estimation pipeline after addressing these challenging problems. The pipeline consists of three major steps: first, we capture antenna photographs remotely using a mobile phone with an IMU including the magnetometer despite the fact that an IMU is indeed composed of only inertial sensors (i.e., accelerometers and gyroscopes) in a strict way; then, we estimate the pose of the antenna relative to the camera from the images with our coarse-to-fine visual pose estimation method, and we estimate the orientation of the IMU relative to the Earth from the IMU outputs; and finally, the downtilt and azimuth angles of the antenna are calculated by concatenating the two poses with the refined transformation between the camera and the IMU as a result of our camera-IMU calibration method.

Accordingly, our major technical contributions include the following:

1. We present an accurate solution to the downtilt and azimuth estimation problem for antennas based on multi-view antenna images and IMU data captured by a mobile phone. To the best of our knowledge, this is the first report of a non-contact method of measuring the pose of an antenna using a mobile phone.
2. We enhance existing camera-IMU calibration models by introducing additional degrees of freedom (DoFs) between the accelerometer and magnetometer, and we define a new error metric based on both the downtilt and azimuth errors instead of a single unified rotational error. This enables us to propose a new camera-IMU calibration method that permits simultaneous improvement of the estimation accuracy for both the downtilt and azimuth angles, making it suitable for tasks in which both types of error are crucial.
3. We propose an automatic method of determining an approximate pose from multi-view antenna contours for visual pose estimation, and we also provide bounds on the search space for pose refinement, thereby converting the underlying unconstrained optimization problem to a constrained one to allow solutions to be obtained with better accuracy.

The paper proceeds with a review of related works in Section 2. A formal formulation of the problem and an overview of our estimation approach are presented in Section 3, and the details of the implementation are given in Section 4 and Section 5. Section 6 describes evaluations of the proposed approach using both synthetic and real-world datasets. Section 7 discusses and concludes the paper with indications of our future work.

## 2. Related Work

There is a vast amount of literature related to pose estimation problems, and the most important and most closely related studies are those concerning visual pose estimation and visual-inertial fusion. We will focus on techniques that specifically address visual pose estimation of rigid objects with known geometries and sparse textures, as well as techniques for camera-IMU calibration, which is a key component of visual-inertial fusion.

### 2.1. Visual Pose Estimation

The general problem of visual pose estimation has been a long-standing topic in computer vision (see [20] for an early review). By adopting an antenna geometry model, we are formulating the problem as one of a 2D-3D matching in which “3D objects are observed in 2D images” [20], the goal of which is “to estimate the relative position and orientation of a 3D object to a reference camera system” [20].

There are two major paradigms for approaching this problem, distinguished by how correspondences are established between the model and the imagery. One is the feature-based approach, in which an image is abstracted into a small number of key-point features. The other is the direct approach, in which image intensities are used directly to determine the desired quantities.

The feature-based approach is typically the most popular solution. The core underlying idea is to compute a set of correspondences between 3D points and their 2D projections, from which the relative position and orientation between the camera and target can then be estimated using various algorithms, such as those for solving the perspective-n-point (PnP) problem [21]. Consequently, the performance of this approach hinges on whether enough features can be detected and correctly matched. Although numerous feature detection and tracking schemes [22,23,24,25] have been developed, these methods are unsuitable for textureless objects. Recently, line features, such as the bunch of lines descriptor (BOLD) [26], have been proposed for handling textureless objects, but on very simple shapes with too few line segments and little informative content, they are still prone to failure. Furthermore, the question of how to build stable 2D-3D correspondences is a topic that is still under investigation.

The direct pose estimation approach attempts to avoid issues of feature tracking and matching by matching model projections to 2D images as a whole. There exists a large class of methods based on template matching. Hinterstoisser et al. proposed a series of template-matching-based methods using inputs based on the distance transform [27], dominant gradient orientations [28] and the recently developed concept of gradient response maps (GRM) [29]; Liu et al. [30] used edge images and included edge orientations in templates in their fast directional chamfer matching (FDCM). GRM and FDCM are state-of-the-art template matching methods. Once an object is registered using a pre-built template, a refinement process, which is usually based on the iterative closest point (ICP) algorithm [31], is performed to refine the object’s pose, such as in FDCM. In 2015, Imperoli and Pretto proposed the direct directional chamfer optimization (D2CO) [32] for pose estimation, in which a non-linear optimization procedure (the Levenberg–Marquardt algorithm) is applied in the refinement stage instead of an ICP-based method, and in a comparison with four ICP-based refinement methods (including FDCM), D2CO demonstrated an advantage in terms of the correct model registration rate. The idea of optimizing the pose parameters has also been pursued in tracking [33] and simultaneous localization and mapping (SLAM) applications [34]. As an alternative to the template matching framework, Prisacariu and Reid [35] introduced a level-set-based modeling method based on a cost function describing the fitness between the estimated pose and the foreground/background models, and they solved the optimization problem using a simple gradient descent approach given an initial pose. Their Pixel-Wise Posteriors for 3D tracking and segmentation (PWP3D) method has been widely used in tasks of simultaneous segmentation and pose estimation, and as a subsequent improvement to PWP3D, Zhao et al. [36] proposed a boundary term to PWP3D (BPWP3D) , which offers finer boundary constraints for more challenging detection environments. However, these (local) optimization-based methods depend on the initial parameters and may become trapped in local optima.

Our pose estimation method predominantly belongs to the second category. By exploiting a shape prior for an antenna and matching its geometric features, we automatically find an initial pose to avoid potential human interaction and any overhead incurred for the building and matching of templates. Moreover, in the subsequent pose refinement step, we construct bounds on the pose search space to transform the original unconstrained optimization problem into a bounded one, which is then solved using global optimization techniques.

Recently, depth cameras have begun to be used for 3D pose estimation. However, current consumer-level depth cameras are not capable of detecting objects at long distances. For example, the maximum detection distance for a Kinect v2 is 4.5 m. Therefore, such approaches have limited applicability to our problem.

### 2.2. Camera-IMU Calibration

To relate measurements in the camera frame to the Earth frame, the relative pose between the camera and the IMU (i.e., the rigid transformation between the two frames) should be known. The process of determining this transformation is usually referred to as camera-IMU calibration [37].

Fleps et al. [38] classified the existing approaches into two categories: approaches that require specialized measurement setups and facilitate closed-form solutions and filter-based approaches with approximate solutions. Mair et al. [39] categorized the approaches into three classes: methods with closed-form solutions, Kalman-filter-based methods and methods that make use of optimization techniques. Here, we offer a review from another perspective, based on the hardware configurations used in the various calibration methods, leading to two groups.

Methods in the first group rely on the gyroscope in an IMU. A prevalent practice is a filter-based approach in which the calibration parameters are integrated into the state vector of an IMU motion filter (e.g., [40,41] (see [42] for an overview)) and are solved simultaneously with other motion states. However, as noted by Maxudov et al. [43], a long state vector naturally imposes certain limitations on accuracy. Moreover, the filter-based framework is unnecessary for offline calibration; based on this insight, Fleps et al. [38] modeled the calibration problem in a non-linear optimization framework by modeling the sensors’ trajectory. In these methods, the camera is in constant motion, and over-simplifying the model of a camera on a mobile phone by using a global-shuttered model instead of a rolling-shuttered model may cause problems, as revealed in more recent works [44,45].

The methods belonging to the other group considered here are also suitable for use with gyroscope-free IMUs. These methods are closely related to hand-eye calibration, or, more concretely, eye-in-hand calibration, an approach used in the robotics community in which the relative pose between the camera and a rigid rig is sought. Since it was first proposed by Shiuand Ahmad in 1989 [46], hand-eye calibration has been largely considered a solved problem (see [47] for a review), and recent research has mainly focused on the development of more powerful solvers [48]. In camera-IMU calibration, the role of the “hand” is played by an accelerometer or an accelerometer-magnetometer pair. In the first complete camera-IMU calibration procedure, proposed by Lobo and colleagues [37], the authors estimated the rigid rotation between the camera and accelerometer as a standalone step in which the rotation was estimated by having both sensors observe the vertical direction in several poses. The camera relies on an ideally vertically placed checkerboard and the accelerometer on gravity to obtain a vertical reference. Their work was released as a [49] toolbox and is widely used. In Vandeportael’s work on a camera that knows its orientation (ORIENT-CAM) [50], a similar idea was applied. However, since the IMU used in ORIENT-CAM consists of an accelerometer and a magnetometer, the relative rotation is estimated by aligning observations of the Earth frame from the IMU and the camera by means of a checkerboard that is ideally laid out such that it is both perfectly horizontal and perfectly northward-oriented. This method requires a carefully placed reference, as in [37], and any error during setup directly introduces bias into the calibration results. Under the assumption of negligible camera translations during the calibration process, in their work on ego-motion [51], Domke and Aloimonos solved for the rotation between the camera and accelerometer by relating gravity observations in IMU frames with the motion of the camera. By considering the relative rotations between different camera frames, they avoided the need for artificial references requiring a rigorous setup.

Our calibration method belongs to the second category. Unlike existing approaches, we consider the difference in precision between the two IMU sensors in a mobile phone and use a finer-grained error metric consisting of two terms, instead of a unified one (as in [50,51]), to reflect the resulting effect. Moreover, we do not assume perfect accelerometer-magnetometer alignment during the assembly of the sensor hardware and thus are able to decouple the accelerometer-related error and the magnetometer-related error. The reasons that we do not adopt a method of the first category are as follows: (1) the dynamic features of the gyroscope and the moving camera are nonessential to our measuring problem, in which instantaneous sensor outputs are employed; and (2) a calibration method that is independent of the gyroscope is applicable to a wider range of devices.

## 3. Problem Formulation and Method Overview

Our goal is to estimate the antenna pose in the Earth frame from multi-view data, which consist of multi-view images of the antenna and IMU (accelerometer and magnetometer) measurements recorded at the exact same instant as each image capture. Below, we first formally define the problem and then present an overview of our solution.

### 3.1. Notation and Problem Formulation

As described in the Introduction, the number of different antenna types in use is quite limited, and therefore, it is reasonable to assume a known 3D antenna geometry once we have identified the antenna type from the acquired images. Let this geometry be denoted by M, and let us assume that the bounding box of the model is centered at the origin point of the object frame (*OF*) and that its three axes are aligned with the axes of *OF*, without loss of generality.

In each of the multi-view images, the antenna (treated as the foreground) is represented by a contour expressed as a list of connected points, denoted by $\Phi_{i},i=1,2,...,P,$ where *P* is the number of viewpoints. Such contours can be the outputs of a procedure based on image segmentation, shape detection or human interaction during image capture; we do not discuss this procedure here. This representation discards any textural information and interior shape information for an antenna, making it generally impossible to obtain a unique pose solution from a single viewpoint. Nevertheless, we opt to simply ignore these two kinds of information because of their instability, as demonstrated in Figure 1. Instead, contours captured from multiple viewpoints enable the determination of a unique solution.

The 3D mesh M and the 2D images are related by camera projections. We model the phone camera as a pin-hole camera, which maps M first from *OF* into the camera frame (*CF*) via an unknown rigid transformation ROC,TOC and then into the image plane via a perspective function. The projective function is determined by a set of intrinsic parameters of the camera, denoted by *K*, which is taken to consist of known constants for a pre-calibrated camera.

In addition to the images, the other important half of the multi-view data consists of the IMU measurements, which encode the orientations of the IMU in the Earth frame (*EF*) when the images were captured. The directions of gravity and magnetic north at a given point on Earth define *EF* in that location, and the accelerometer and magnetometer sensors of the IMU respond to the gravitational force and magnetic flux, yielding their projections onto the sensor axes. We let $S_{i}=\left(a_{i},\;m_{i}\right),i=1,2,\ldots ,P$, denote the overall IMU measurements, where $a_{i}$ denotes an accelerometer measurement and $m_{i}$ denotes a magnetometer measurement.

To ensure an accurate formulation of the problem, there are two small misalignments that we should consider. First, the accelerometer frame, denoted as *A*, should ideally coincide with the magnetometer frame, denoted as *M*, such that the orientation of the IMU in *EF* can be determined from the outputs of these two sensors (e.g., [52]). However, because the sensor hardware usually resides on different chipsets in a mobile phone, a small rotation may exist between the two sensor frames. Moreover, other environmental factors (especially magnetic factors) may affect the rotation of each sensor frame, thereby worsening the misalignment. Let this unknown rotation be denoted by RMA, which we can then use to re-align *A* and *M* once it is known. For convenience, we also regard *A* as the overall IMU frame (*IF*) when doing so will not cause confusion. Second, the frames of the camera module and IMU module in a mobile phone should also ideally be perfectly aligned, differing only by exchanges in the directions of the axes, which is also generally not true in reality. We describe the true relation as a rigid transformation (RCA, TCA) (or (RCI, TCI)), and although it is easy to obtain an approximation of the relative pose between the camera and IMU frames from the mobile phone API, finding the precise transformation requires greater effort. For the pose estimation problem, we temporarily take these two misalignments as priors.

Our final goal in the estimation problem is to determine the antenna’s downtilt and azimuth angles in the Earth frame, which together represent the rotation of the antenna relative to *EF*, denoted by ROE. The symbol of *O* represents the object coordinate frame of the antenna.

**Figure 2:**
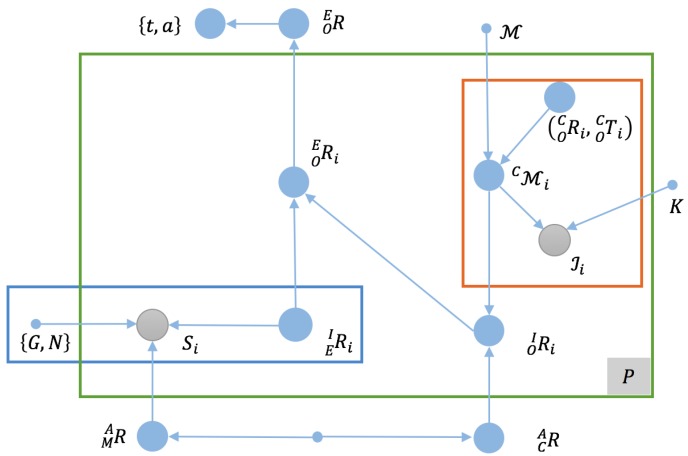
The antenna pose estimation problem represented as a graph model. Filled blue circles represent unknown quantities; filled gray circles represent known quantities; solid dots represent priors; the green-boxed region represents the capture of *P* groups of data; and the arrows indicate the dependencies between the quantities.

We summarize these quantities and their relationships in the graph model shown in Figure 2. A straightforward interpretation of the graph model provides us with a formulation of an estimation problem with a conditional cost function given all priors and observations, which is:

(1) fROE|RMA,RCA,K,M,S1,S2,…,SP,Φ1,Φ2,…,ΦP,

where ROE is the pose to be estimated.

The two main sources of input are the camera projection process and the IMU sensing process, so we re-express Equation (1) as follows:

(2a) f=∑i=1Pg1ROCi|K,M,Φi+g2REAi|Si,RMA=∑i=1Pg1ROCi|K,M,Φi+∑i=1Pg2REAi|Si,RMA,

(2b) s.t.ROE=RAEiRCAROCi,

where $g_{1}$ is the projection-related error and $g_{2}$ is the sensing-related error; the constraint Equation (2b) models the relation between *CF* and *IF* and thus relates two error terms. Note that Equation (2a) is a generic formulation of our pose estimation problem in the Earth frame, and different solutions may arise depending on the choices of $g_{1}$ and $g_{2}$.

### 3.2. Method Overview

A direct optimization-based solution to Equation (2a) is impractical because of its high dimensionality; therefore, we will break the problem down into smaller parts to solve it.

Referring to the original graph model presented in Figure 2, we find that the first item in Equation (2a), which corresponds to the red-outlined region in the upper right of the figure, describes *P* model-based visual pose estimation problems, and similarly, the second item, corresponding to the blue-outlined region in the lower left of Figure 2, describes the IMU orientation estimation problem seeking the rotation of IF in EF denoted by RIEi, for which effective solutions (e.g., [52]) are available under the assumption that we have already aligned $A^{i}$ and $M^{i}$ via RMA.

With these insights, given that RCI, ROCi and RIEi are available, using the graph model to determine ROE becomes a simple process of passing messages through a chain, as follows:

ROE=avgROEi=avgRIEiRCIROCi,

where avg is used to fuse estimations from multiple viewpoints. Using ROE, we can calculate the downtilt and azimuth angles of the antenna.

In addition, we note that the priors RCA and RMA (i.e., the relative poses between the camera, accelerometer and magnetometer) are inherent to each specific mobile phone; thus, they need to be calculated only once and can then be stored for later use. To acquire the exact values of these rotations, we employ a dedicated offline camera-IMU calibration process, which will be described in Section 4.

To summarize, our solution for antenna pose estimation in the Earth frame consists of the following four main steps:

1. For a given phone, we compute the relative poses between its camera and IMU sensors using an offline camera-IMU calibration procedure. Once calculated, the relative poses of the camera and IMU sensors will not change during the antenna pose estimation process.
2. Using the antenna model and images obtained from calibrated viewpoints, we estimate the relative pose between the antenna frame and the camera frame for each viewpoint.
3. We correct the IMU data using the relative rotation between the accelerometer and magnetometer from (1), and we calculate the rotation of the IMU in the Earth frame for each viewpoint using existing IMU orientation estimation techniques.
4. We concatenate the antenna pose in the camera frame and the IMU orientation in each viewpoint with the relative camera-IMU rotation from (1) to obtain the antenna rotation in the Earth frame; antenna poses in all viewpoints are averaged to calculate the resulting downtilt and azimuth angle.

**Figure 3:**
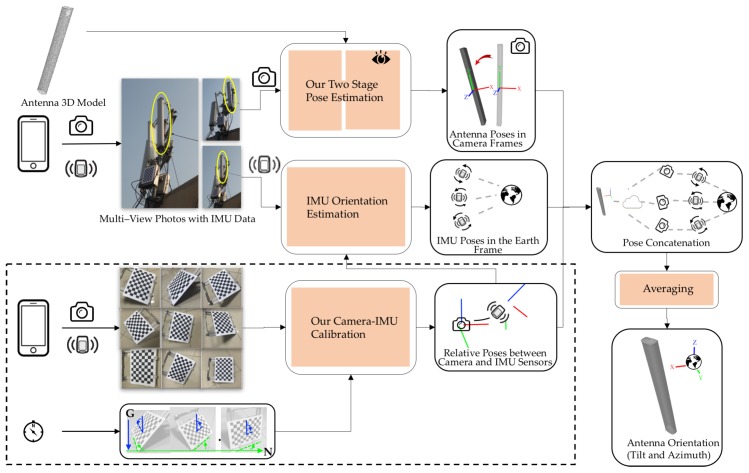
Overview of the proposed method. Antenna images captured from multiple viewpoints are used to estimate the antenna pose in the camera frames based on the 3D antenna model. By correlating the IMU orientations in the Earth frame with the camera orientations, the antenna pose in the Earth frame is computed by averaging the poses estimated from all viewpoints. The camera-IMU calibration provides important information on the relative sensor rotations for improving the IMU orientation estimation and the visual-inertial correlation.

Figure 3 provides an overview of our method. The two key elements of our method are the determination of RCA and RMA in Step (1) and the estimation of ROV in Step (2). We describe the corresponding procedures in detail in Section 4 and Section 5.

## 4. Relative Poses between the Camera and IMU Sensors

In this section, our aim is to accurately determine the relative rotations between the camera, accelerometer and magnetometer to improve the accuracies of downtilt and azimuth estimation for a remote target.

We use a checkerboard to capture the data we need for calibration. The board is placed in several orientations, and for each placement, we measure the downtilt and azimuth angles and capture multi-view data in the same manner used for capturing data from an antenna. This checkerboard pose measurement step replaces the careful checkerboard setup required in [37,50]. Multiple groups of data are captured to provide sufficient constraints for the calibration.

**Figure 4:**
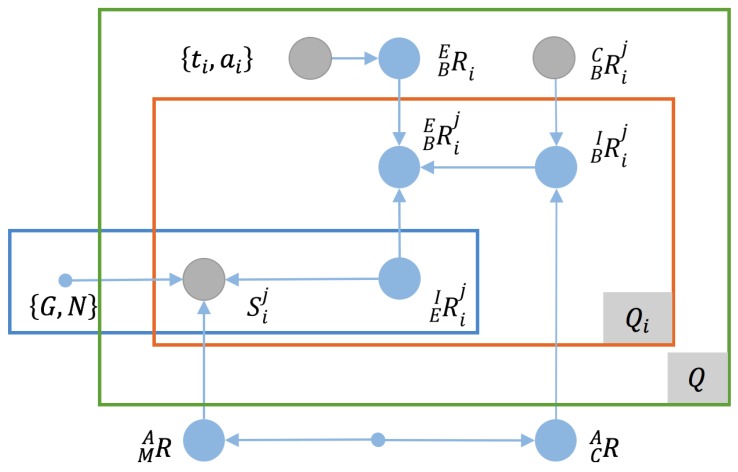
The camera-IMU calibration problem represented as a graph model.

Suppose that we have a group of calibration data that consists of *Q* checkerboard placements with measured downtilt and azimuth angles of $\left(t_{i},\;a_{i}\right),i=1,2,\ldots ,Q,$ and that multi-view data have been captured from $Q_{i}$ viewpoints for the *i*-th checkerboard placement. Then, we can model the calibration using a graph model similar to that presented for the antenna pose estimation problem, as shown in Figure 4.

Unlike in the case of the antenna pose estimation problem, because of the maturity of camera calibration techniques (e.g., [53]), the relative pose between the camera and the checkerboard is considered to be known, and the downtilt and azimuth angles of the checkerboard are regarded as the ground truth. Thus, we can transform the graph model into the following optimization problem:

(3) argminRCA,RMA∑i=1Q1Qi∑j=1QiδRREBi,RCBijRICREIijs.t.haREBi=ai,htREBi=ti,

where $\delta_{R}$ is a distance function or metric for rotations, which we will explain in detail later, and REIij is the IMU orientation in *EF* as calculated from $S_{i}^{j}$, which is the *j*-th frame of sensor data in the *i*-th group of calibration data, with existing methods like [52], after the accelerometer and magnetometer measurements have been aligned via RMG. The symbol of *B* represents the coordinate frame of the checkerboard.

**Figure 5:**
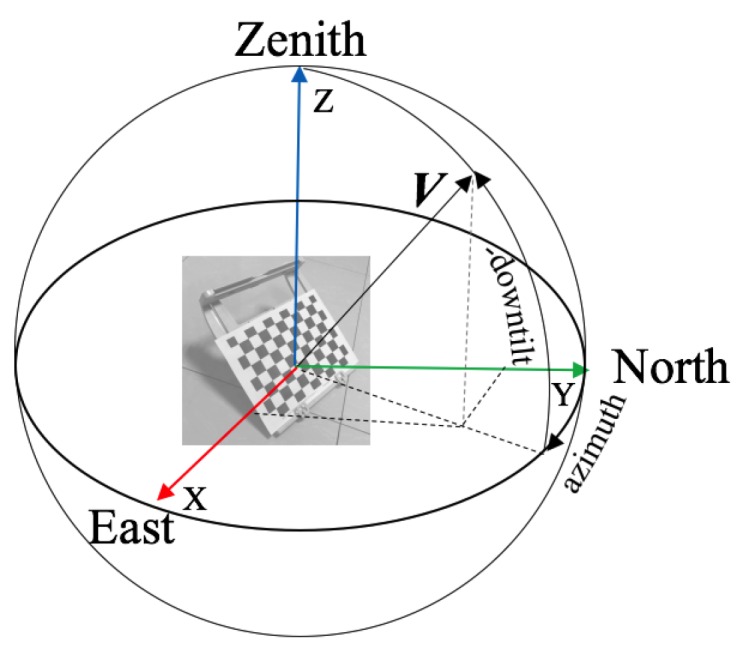
Illustration of the definitions of the (checkerboard) downtilt and azimuth angles in the Earth frame where the X, Y and Z point towards east, north and zenith. The direction normal to the front surface of the checkerboard is used to define both its (down)tilt and azimuth angles in this example.

The functions of $h_{t}$ and $h_{a}$ are defined for calculating the downtilt and azimuth angles of the checkerboard. In astronomy, for a vector V in *EF*, its tilt and azimuth angles are defined as follows:

$$\left\{\begin{array}{c} t=arcsin\left(V(2)\right)\\ a=arctan\left(\frac{V\left(0\right)}{V\left(1\right)}\right), \end{array}\right.$$

where *t* is the tilt angle, *a* is the azimuth angle and $V\left(i\right)$ is the *i*-th component of V. For a checkerboard, we can use its edge directions, its surface normal direction or a combination thereof to describe its tilt and azimuth angles, and since most antennas are pointing downwards, we prefer to use the term downtilt instead of tilt, which are opposite from each other. To be specific, suppose that we choose a direction *v* on the checkerboard to define the downtilt angle and that the rotation of the checkerboard relative to *EF* is RBE; then, the downtilt angle of the checkerboard in *EF* is defined by RBE·v. For convenience, we denote the above process by the function htRBE, where we omit any reference to a predefined v. We can formally define the azimuth angle of the checkerboard in a similar manner and encode the process as htRBE. An illustration is presented in Figure 5.

**Figure 6:**
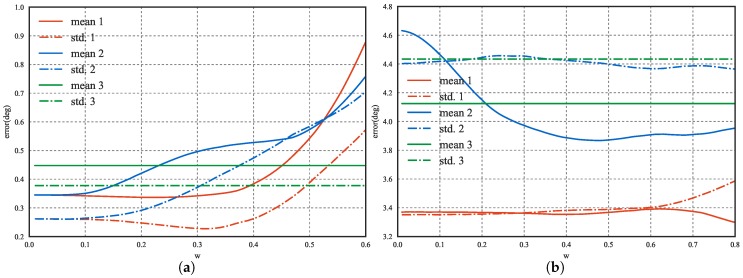
Effects of the weighting parameter on downtilt (**a**) azimuth (**b**) estimation for three calibration configurations (best viewed on screen): (1) no calibration, (2) calibration without accelerometer-magnetometer DoFs and (3) calibration with accelerometer-magnetometer DoFs.

In Equation (3), the introduction of RMG, i.e., the relative rotation between the accelerometer and magnetometer, is a key element that differentiates our method from previous camera-IMU calibration methods. We have explained our motivations for this in Section 1, and further evidence supporting our approach is provided by the contrasting behaviors of the downtilt and azimuth error curves with and without the additional DoFs, as shown in Figure 6, which illustrates that it is difficult to find a balance such that both the downtilt and azimuth errors can be kept simultaneously low when RMG is ignored.

To complete our definition of Equation (3), we design $\delta_{R}$ to be a rotation metric defined in terms of the downtilt and azimuth angles, such that, for two orientations $R_{1}$ and $R_{2}$, we have:

(4) $$\begin{array}{cc} \delta_{R}\left(R_{1},R_{2}\right) & =\delta_{R}^{\prime }\left(\left(h_{t}\left(R_{1}\right),h_{a}\left(R_{1}\right)\right),\left(h_{t}\left(R_{2}\right),h_{a}\left(R_{2}\right)\right)\right)\\ & =\left(1-w\right)\delta_{t}{\left(h_{t}\left(R_{1}\right),h_{t}\left(R_{2}\right)\right)}^{2}+w\delta_{a}{\left(h_{a}\left(R_{1}\right),h_{a}\left(R_{2}\right)\right)}^{2};0<w<0.5, \end{array}$$

where $\delta_{R}^{\prime }$ is a rotation distance function defined in terms of the downtilt and azimuth angles, $\delta_{t}$ and $\delta_{a}$ are two special functions for calculating the minimal differences in the downtilt and azimuth angles based on their periodicity and *w* is a weighting parameter that will be explained later. A simple choice for $\delta_{t}$ and $\delta_{a}$ is the Euclidean distance after the transformation of the angles into the same phase.

Note that our metric is defined based on the downtilt and azimuth angles and thus has only two DoFs, meaning that it is an incomplete representation of a rotation. Although it would be easy to add another DoF to the definition, we choose not to do so to decrease the number of measurements needed during data capture.

We include the weight parameter *w* in the final expansion in Equation (4) to reduce the effect of the azimuth-related error on the overall cost. As is known from [52], the downtilt reading of an IMU relies solely on the accelerometer output, whereas the heading (azimuth) measurement predominantly depends on the magnetometer output. However, the precision of the accelerometer in a mobile phone is typically much higher than that of the magnetometer, and the magnetic environment is highly unstable compared to the gravitational environment in practice. Hence, the scales of the errors on the two components of $\delta_{R}$ are likely to be unbalanced, which may lead to non-optimal solutions for the overall calibration; by restricting *w* to a value less than 0.5, we can re-balance the two types of errors.

Although we cannot determine *w* analytically, we can show that the calibration accuracy is insensitive to *w* when the value of *w* is sufficiently low, as seen from the experimental results presented in Figure 6. Empirically, we recommend keeping this value in the range of [0.1,0.3].

Combining Equations (3) and (4), we obtain:

(5) argminRCG,RMG∑i=1Q1Qi∑j=1Qi1−wδtti,htRCBijRICREIij2+wδaai,haRCBijRICREIij2;0<w<0.5.

Equation (5) is written in a standard least-mean-square form, and it can be effectively solved using the Levenberg–Marquardt algorithm [54].

## 5. Antenna Poses Estimated from Captured Images

Considering that the scene containing the antenna is static from one viewpoint to another, if we insert a camera calibration object (e.g., a checkerboard) into the scene and employ a suitable extrinsic camera calibration technique (e.g., [53]) or apply a structure-from-motion (SfM) technique (e.g., [55]) to the background, we can obtain the relative poses of the camera corresponding to all viewpoints relative to a visual reference frame, meaning that the task can be formulated as a visual pose estimation problem using data from *P* viewpoints. Let the viewing reference frame (*VF*) be denoted by *V*; let the camera frames (*CF*s) be denoted by $C_{i},i=1,2,\ldots ,P$; and let the relative poses be denoted by RVCi,VCTi,i∈1,2,…,P. Then, we need to find only the relative pose between *O* and *V* instead of the original 3P unknowns. This process is expressed as follows:

(6) ∑i=1Pg1(ROIi|K,M,Φi)=g1′(ROV|K,M,Φ1,Φ2,…,ΦP),

where $g_{2}^{\prime }$ describes the error on the visual pose estimation based on images acquired from *P* viewpoints.

To complete our definition of Equation (6), we define $g_{1}^{\prime }$ as a contour-based distance function between the projections of the 3D antenna model and the antenna foregrounds in the real images:

(7) argminROV∑i=1PdCΦiπROV,TOV,Φi,

where ΦiπROV,TOV is the projection contour of the antenna in pose (ROV,TOV) from the *i*-th viewpoint of extrinsic camera parameters of (RVCi,TVCi); and $d_{C}\left(\cdot \right)$ is a contour-based distance function.

Our approach does not rely on any assumption regarding the form of $d_{C}$. Without loss of generality, we define $d_{C}$ based on a point-to-contour distance:

$$d_{C}\left(\Phi^{\prime },\Phi^{\prime \prime }\right)=\sum_{X\in \Phi^{\prime }}d_{p}^{c}\left(X,\Phi^{\prime }\right)+\sum_{X\in \Phi^{\prime \prime }}d_{p}^{c}\left(X,\Phi^{\prime \prime }\right),$$

where $\Phi^{\prime }$ and $\Phi^{\prime \prime }$ are the two contours to be matched and $d_{p}^{c}$ is the operator for calculating the shortest Euclidean distance between a point and all points on a contour:

$$d_{p}^{c}\left(X,\Phi \right)=\min_{Y\in \Phi }\left({\left|X-Y\right|}^{2}\right).$$

Another way to interpret $d_{p}^{c}$ is to treat it as an embedding function of the level set underlying a contour; for details, we refer the reader to [56]. An efficient algorithm to compute $d_{p}^{c}$ is given in [57].

**Figure 7:**
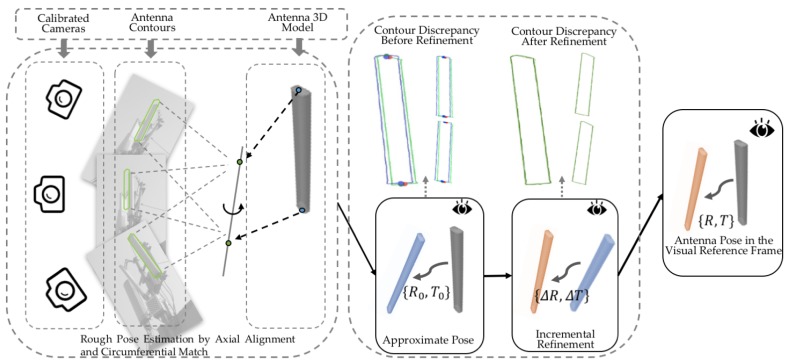
As shown in the leftmost box, the position of the 3D principal axis is recovered from multi-view contours (green), and the 3D antenna model (gray) is aligned with the recovered axis and rotated to find a coarse estimate of the antenna pose. As shown in the left column of the middle box, there are discrepancies between the antenna contours and the projections from the approximate pose (blue), and as shown in the right column, the approximate pose can be globally refined to reduce the contour discrepancy with respect to the resulting refined pose (pink). In the rightmost box, the final overall estimation result is shown as the transformation from the model frame (gray) to the reference frame (pink).

To solve Equation (6), we adopt a coarse-to-fine strategy. First, we exploit the fact that most antennas are approximately cuboid in shape to recover an approximate pose, by aligning the 3D principal axis of the model with the 2D principal axes in the multi-view images and finding a proper rotation around the principal 3D axis. Then, based on this coarsely estimated pose, we construct bounding constraints to be applied to the pose search space, thereby allowing us to seek the optimal pose by using global optimization techniques to minimize Equation (6). An overview of our approach is provided in Figure 7.

### 5.1. Approximate Pose Estimation for Initialization

#### 5.1.1. Axial Alignment

**Figure 8:**
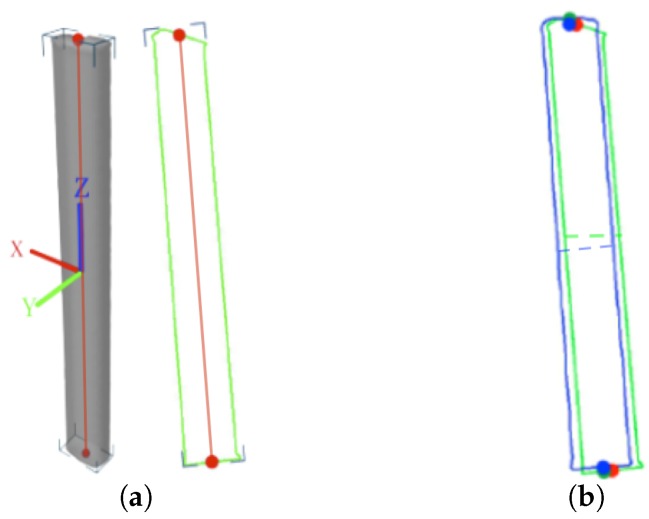
Axial alignment. (**a**) The 3D/2D principal axes detected using a rotated bounding box; (**b**) projections of endpoints of model principal axis and recovered 3D axis are shown as the blue and red dots, and the endpoints of the detected 2D principal are shown as the blue dots. Note that some misalignment between them may exist.

The strong axiality of the antenna shape originates from the fact that most directional antennas are approximately cuboid in shape. We use the concept of principal axes to describe the axiality of both the antenna model and the antenna projections in images. We define the 3D principal axis of the model as the 3D line segment that crosses the centroid of the model, is oriented in the direction along which the model extends the farthest and is bounded by the mesh (as illustrated in Figure 8a); similarly, the 2D principal axis of a projection is the 2D line segment that crosses the centroid of the 2D silhouette, is oriented in the direction along which the silhouette extends the farthest and is bounded by the contour (as illustrated in Figure 8a).

The first step of our coarse pose estimation procedure is to find a pose for which the 3D and 2D axes are aligned. First, we detect the 2D/3D principal axes from the images and the 3D model. There are many ways to achieve this, for example, by applying principal component analysis (PCA), or independent component analysis (ICA) to the contour points and the 3D model vertices, or by finding the (rotated) bounding box of the contour/model.

Once the 2D axes have been found for all viewpoints, we recover the 3D principal axis in *VF* from the end points of the 2D axes using triangulation methods (e.g., [58]). Let the recovered 3D axis be denoted by $\vec{E_{0}^{\prime }E_{1}^{\prime }}$, and let the 3D principal axis of the model be denoted by $\vec{E_{0}E_{1}}$; then, we have:

(8) $$R_{⊥}^{\prime },T^{\prime }=\underset{R,T}{argmin}\sum_{k=1,2}{\left|RE_{k}+T-E_{k}^{\prime }\right|}^{2},$$

where $R_{⊥}^{\prime },\;T^{\prime }$ is the pose to be estimated. Equation (8) is also known as the generalized Procrustes problem and can be efficiently solved analytically [59].

#### 5.1.2. Circumferential Match

However, the solution to Equation (8) is not unique: from a geometric point of view, $R_{⊥}^{\prime }\;$ describes only the yaw and pitch of the antenna. Let the two angles be $\alpha^{\prime }$ and $\beta^{\prime }$; we can write $R_{⊥}^{\prime }$ as $R_{⊥}^{\prime }\left(\alpha^{\prime },\beta^{\prime }\right)$. The left roll angle, which describes the rotation around $\vec{E_{0}^{\prime }E_{1}^{\prime }}$, is still undetermined.

**Figure 9:**
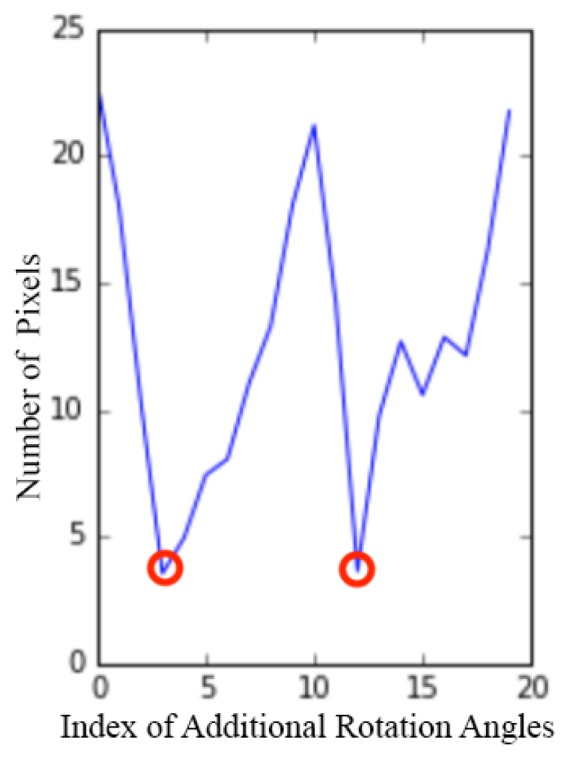
Removal of the rotational uncertainty about the (recovered) 3D principal axis. The antenna model is rotated around the recovered principal axis with a fixed step size (X axis), and the average difference between the antenna widths at the center in the projections and the real images (Y axis) is treated as the cost that represents the degree of closeness between the current pose and the ground truth. Two minima are clearly observed.

To eliminate the remaining uncertainty, we enumerate the discrete rotations of the model around $\vec{E_{0}^{\prime }E_{1}^{\prime }}$ based on $R_{⊥}^{\prime },T^{\prime }$ to find a rotation that minimizes the difference between the widths of the antenna silhouettes at their centers in the real images and in the projections (as indicated by the dashed lines in Figure 8b). Figure 9 shows an example of how the width difference changes with the rotation; two minima are observed because of the symmetry of the antenna model, and we select the correct one based on prior knowledge of which side of the antenna is facing the camera.

Let the best rotation angle determined through enumeration be $\gamma^{\prime }$, and let the additional rotation it represents be denoted by $R_{∥}^{\prime }\left(\gamma^{\prime }\right)$; then, by combining $R_{⊥}^{\prime }$ and $R_{∥}^{\prime }$, we obtain the following approximate rotation:

$$R\left(\alpha^{\prime },\;\beta^{\prime },\gamma^{\prime }\right)=R_{∥}^{\prime }\left(\gamma^{\prime }\right)R_{⊥}^{\prime }\left(\alpha^{\prime },\beta^{\prime }\right).$$

Figure 8b shows an example of how the contours from a real image and from the antenna projection based on the recovered pose are aligned for a single viewpoint.

### 5.2. Pose Refinement

#### 5.2.1. Bounds on Pose Parameters

The pose obtained above is inaccurate as a result of three factors: (1) displacements between the detected 2D/3D principal axes and their true positions; (2) potential errors in the triangulation of the 3D principal axis from the images; and (3) imprecision in the determination of the roll angle. Consequently, we wish to further refine this pose.

A simple approach is to treat the approximate pose as an initialization and then iterate until convergence is achieved, as done in PWP3D [35] and D2CO [32]. Although our approximated poses function well as initializations in most cases, they still cannot guarantee the avoidance of local minima. For higher accuracy, we attempt to find bounds on the pose search space that will allow us to use global optimization techniques to solve Equation (6).

**Figure 10:**
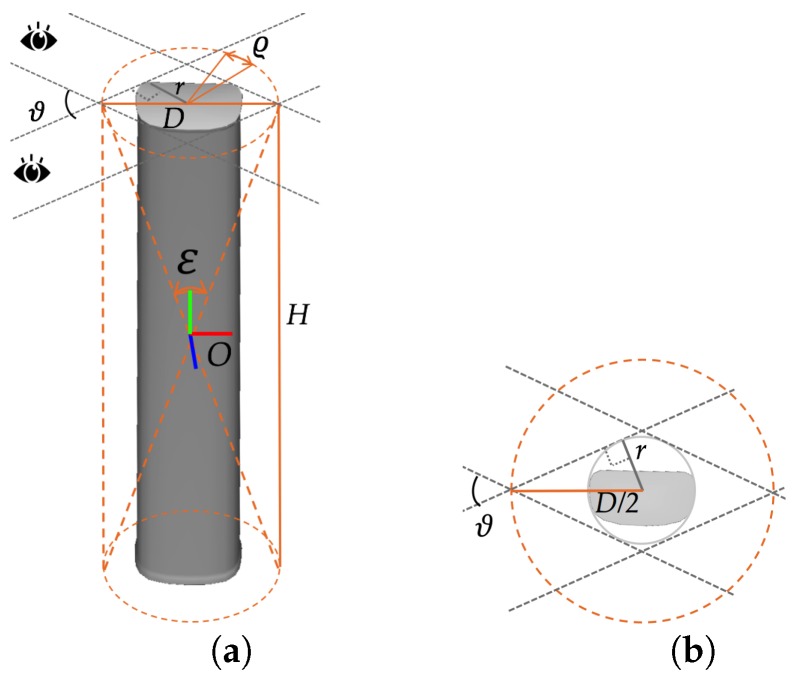
Constraints on the refinements of rotation and translation. (**a**) The double cone defines the rotation constraints, and the cylinder defines the translation constraints; (**b**) the radius of the cone is related to the bottom size of the antenna and the angle between two most distant viewpoints.

We first concentrate on the rotational component of the pose. The first two sources of imprecision are predominantly related to the yaw and pitch of the antenna. We observe that the projection of the 3D principal axis based on the approximate pose never falls out of the area enclosed by the two long side edges of the antenna silhouette for each viewpoint, which means that the recovered 3D principal axis always lies within the double cone enclosing the visual hull of the antenna suggested by contours from multi-view images, as indicated in Figure 10a. Let the viewing angle between the two most distant viewpoints be denoted by ϑ, and let the radius of the bounding cylinder of the antenna be denoted by *r*; then, the diameter *D* of the cone is $2r/\sin\left(\frac{\vartheta }{2}\right)$ (see Figure 10b), and the opening angle ε of the cone is $2\arctan(\frac{D}{H})$, where *H* is the height of the antenna (see Figure 10a). In this way, we can obtain bounding constraints on the refinements to the yaw and pitch. In practice, we have found that the approximate rotation is usually much closer to the true value than these bounds would suggest, so we scale the bounds by an empirical factor $w_{1}$ to further shrink the search space.

Regarding the last source of inaccuracy, i.e., that affecting the roll angle, we already have a natural bound, namely the granularity used when enumerating the roll angle in Section 5.1.2, which we let be denoted by ϱ.

To summarize, the bounded search space for the refined rotation is:

$$\begin{array}{cc} & R\left(\alpha ,\beta ,\gamma \right)=R\left(\alpha^{\prime }+\Delta \alpha ,\beta^{\prime }+\Delta \beta ,\gamma^{\prime }+\Delta \gamma \right)=R_{∥}^{\prime }\left(\gamma^{\prime }+\Delta \gamma \right)R_{⊥}^{\prime }\left(\alpha^{\prime }+\Delta \alpha ,\beta^{\prime }+\Delta \beta \right),\\ s.t.\; & |\Delta \alpha ,\Delta \beta ,\Delta \gamma |<{\left(w_{1}\varepsilon ,w_{1}\varepsilon ,\varrho \right)}^{T}, \end{array}$$

where Δα, Δβ and Δγ describe the difference between the approximate rotation and the real value.

Regarding the translation of the model, it can be similarly observed that the projection of the center of the model always falls within the antenna foreground in the image and is usually not far from its true position. This means that in *OF*, the true translation is confined to the cylinder formed by the top and bottom of the double cone found above (see Figure 10a), yielding bounds of $\left(D,H,D\right)$ on translations in *OF* based on $T^{\prime }$. Moreover, for reasons similar to those motivating the introduction of the scale factor $w_{1}$, we also introduce an empirical factor $w_{2}$ for the translation along the principal axis. Finally, in *VF*, we have the following bounded translation space:

$$\begin{array}{cc} & T=T^{\prime }+\Delta T,\\ s.t.\; & |\Delta T|<R^{\prime }{\left(D,w_{2}H,D\right)}^{T}, \end{array}$$

where the vector ΔT describes the difference between the approximate translation and the real value.

#### 5.2.2. Refinement via Constraint-Based Optimization

To summarize, the optimization defined in Equation (7) is now rewritten as:

(9) $$\begin{array}{c} \underset{\Delta \alpha ,\Delta \beta ,\Delta \gamma ,\Delta T}{min}\sum_{i=1}^{P}d_{c}^{c}\left(\Phi_{i}^{\pi }\left(R_{∥}^{\prime }\left(\gamma^{\prime }+\Delta \gamma \right)R_{⊥}^{\prime }\left(\alpha^{\prime }+\Delta \alpha ,\beta^{\prime }+\Delta \beta \right),T^{\prime }+\Delta T\right),\Phi_{i}\right)\\ \;s.t.\;\left\{\begin{array}{c} -\left(\varepsilon ,\varepsilon ,e\right)<\left(\Delta \alpha ,\Delta \beta ,\Delta \gamma \right)<\left(w_{1}\epsilon ,\;w_{1}\epsilon ,\varrho \right)\\ -R^{\prime }{\left(D,w_{2}H,D\right)}^{T}<\Delta T<R^{\prime }{\left(D,w_{2}H,D\right)}^{T}. \end{array}\right. \end{array}$$

The constraints expressed in Equation (9) are simple box-shaped boundary constraints, which enable us to search for the refined pose in a reduced space by seeking global convergence using algorithms such as the dividing rectangles (DIRECT) algorithm [60]. Typically, another round of local optimization (we use constrained optimization by linear approximations (COBYLA) [61]) is then performed to also ensure local optimality.

#### 5.2.3. Validation of the Effectiveness of the Bounds Applied for Pose Refinement

In Table 1, we present the statistics of the estimation error with respect to the ground truth based on the refinement results obtained using the Broyden–Fletcher–Goldfarb–Shanno (BFGS) algorithm [62] and COBYLA as solvers for Equation (9) on an antenna dataset named AntennaL, which consists of 65 groups of data (described in detail in Section 6.1). Both solvers seek a local optimum, but the latter is a solver that can take advantage of bounding constraints, whereas the former is not. A comparison reveals that COBYLA yields far better estimates of both the downtilt error and the azimuth error, although there are cases in which the azimuth error is greater than the maximum tolerance allowed in the industry (15 degrees).

## 6. Experimental Results

We experimentally evaluated the overall pipeline of the proposed method of antenna pose estimation. The two key steps of camera-IMU calibration and visual pose estimation were evaluated and compared with state-of-the-art methods; the effects of various camera-IMU calibration method and camera extrinsic calibration methods on antenna pose estimation were compared, and the accuracy of the overall pipeline was reported on data of working antennas.

Note that a downtilt deviation of 1.5 degrees will drastically affect the performance of an antenna [19], and the empirical azimuth deviation tolerance is approximately 15 degrees. Therefore, the overall downtilt and azimuth estimation error has to be less than the two values for industrial applications, such as network optimization.

### 6.1. Setup and Datasets

We captured multiple datasets and organized them into two groups for various evaluations. In the first group, there are three datasets, named BoardF, BoardL and Motion, which are for evaluations of camera-IMU calibration methods; in the second group, there are two datasets, named AntennaL and AntennaS, which are for evaluations of visual pose estimation methods and the overall pipeline.

**Figure 11:**
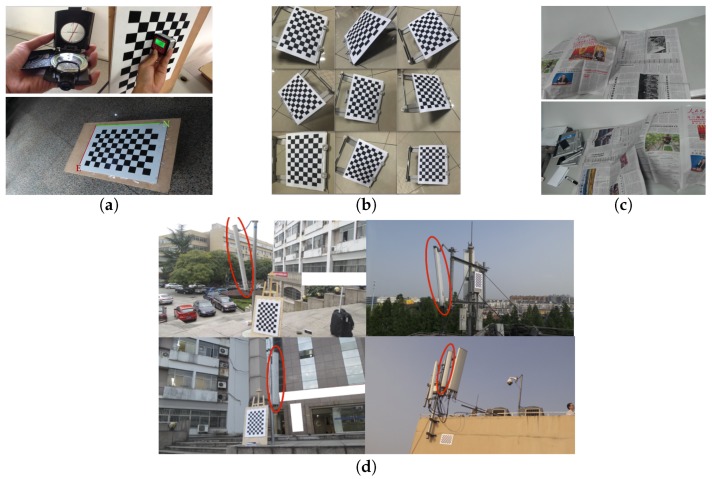
Data capture tools and examples from the datasets. The top part of (**a**) shows the tools used to measure the downtilt and azimuth angles of the checkerboard used for calibration; the bottom part of (**a**) shows an example image from BoardF in which the board is aligned with the Earth frame; (**b**) shows examples of checkerboards in various orientations from BoardL; (**c**) shows two snapshots from data prepared for camera-to-IMU calibration and synchronization toolbox (CRISP) in Motion; (**d**) shows examples from multi-view images of antennas from AntennaL (left) and AntennaS (right).

All of these datasets are captured using a Samsung© Galaxy S4 Zoom smart phone with an Android application that we developed ourselves, and a tripod is used to ensure stable sensor data when necessary. Examples are presented in Figure 11, and details are given below. The datasets are publicly available at http://zju-capg.org/antenna/data.

#### 6.1.1. Camera-IMU Calibration/Evaluation Dataset

The dataset of BoardF consists of multi-view checkerboard data, in which the checkerboard is always laid horizontally with the X or Y axis pointing directly north and multi-view data are captured around the checkerboard about every 15 degrees. The specific placement of the checkerboard satisfies the needs of [63], which requires an ideally vertical or horizontal checkerboard, and the needs of [50], which requires a checkerboard aligned to the north. We organized BoardF into two sub-datasets: originally, the data were captured in a hall and a balcony separately and were therefore divided into two subsets named hall and balcony. Either hall or balcony can be used to perform the camera-IMU calibration; however, we prefer to use them together (i.e., the dataset of BoardF) to avoid potential overfitting.

BoardL was collected for calibration using our method. The restrictions on the checkerboard orientation applied in BoardF are removed, and the downtilt and azimuth angles of the checkerboard are treated as the ground truth.

Both BoardF (including its two subsets) and BoardL are further split in half, with one half serving as the calibration set and the other serving as the evaluation set.

Motion is a dedicated set for use in camera-to-IMU calibration and synchronization toolbox(CRISP) [44], which requires inputs consisting of video data and gyroscope readings. We note that the implementation of CRISP as provided by its author is built using fixed-ratio gyroscope data; however, because the gyroscope in an Android phone works in an event-based manner, we resampled the gyroscope readings as suggested by the author.

#### 6.1.2. Multi-View Antenna Dataset

We collected two datasets of multi-view antenna data, each serving a different purpose. AntennaL consists of 65 groups of data captured on campus using an antenna whose downtilt angle ranged from approximately 1.5 degrees to approximately 11.5 degrees and whose azimuth angle ranged from −180 to 180 degrees, and each group of data consists of three viewpoints. AntennaS is similar to AntennaL, but was collected based on working antennas; however, because of restrictions imposed by the local telecom company, our access was limited to only two antennas, from which we captured six groups of data consisting of three to six viewpoints for each group. A checkerboard was placed in the scene in both AntennaL and AntennaS to enable the determination of the extrinsic camera parameters. We evaluated the accuracy of our method on AntennaL, which contains many more groups of data and antenna poses, and verified the performance of our method in a real environment using AntennaS.

### 6.2. Comparison with State-of-the-Art Methods

#### 6.2.1. Camera-IMU Calibration Methods

In this section, we first compare our method (under two parameter configurations) with those presented in [37,50,51] on BoardF. We calibrated each method on the portion data for calibration in hall, balcony and BoardF and then evaluated them on the corresponding evaluation data. Although we assert that the use of a gyroscope is unnecessary for the estimation task, we also evaluated a recent gyroscope-based camera-IMU calibration method, namely CRISP [44]. Because it requires video and gyroscope input, CRISP was calibrated on Motion.

The calibration methods were evaluated by estimating the downtilt and azimuth angles of the checkerboards by applying the calibration results (i.e., the rotations between the camera and the IMU sensors) to an uncalibrated phone. Statistics are presented in Table 2 as the mean error and standard error with respect to the ground truth.

As seen from Table 2, our method always shows the best or very close to the best performance in downtilt estimation. Meanwhile, for azimuth estimation, none of the three existing methods can reduce the error effectively, whereas our method always achieves a large improvement by considering the possible accelerometer-magnetometer misalignment.

Moreover, we also report the results for the accuracy of our calibration method when it is calibrated on a proper dataset, BoardL, in which no restrictions are placed on the checkerboard orientations. We tested configurations of either keeping or removing the variables describing the relative rotation between the accelerometer and magnetometer in our calibration, as well as varying the weighting parameter *w* from 0.05 to larger values with a fixed step size of 0.01.

In Figure 6, the two solid blue lines (calibration without accelerometer-magnetometer DoFs) for the downtilt and azimuth angles show opposite tendencies: when *w* is low, the downtilt estimation is improved relative to the baseline in green lines, whereas the azimuth error is very large, and when *w* is high, the situation is reversed. When the additional DoFs between the accelerometer and magnetometer are added, both types of error are effectively reduced simultaneously when *w* is below 0.3, as shown by the red lines. This indicates the existence of rotation between the accelerometer and magnetometer.

Furthermore, the curves of the downtilt/azimuth error versus *w* represented by the red lines show broad plateaus, thereby demonstrating the robustness of the method with respect to *w*.

Therefore, we report our method to work best with the additional DoFs between accelerometer and magnetometer and with *w* set to a low value approximately in the range [0.1,0.3]. With these configurations, the downtilt and azimuth accuracies are around respectively 0.35 and 3.4 degrees on average with standard deviations of 0.25 and 3.4 degrees, which are generally superior to those reported by the state-of-the art in Table 2.

#### 6.2.2. Visual Pose Estimation Methods

For a comparison of our visual pose estimation method with PWP3D [35] and D2CO [32], each method was plugged into our overall pipeline for the estimation of the antenna downtilt and azimuth angles, and the estimation results were then evaluated against the ground truth. The camera-IMU calibration parameters were obtained from the results of our calibration method on BoardL with *w* set to 0.1.

**Figure 12:**
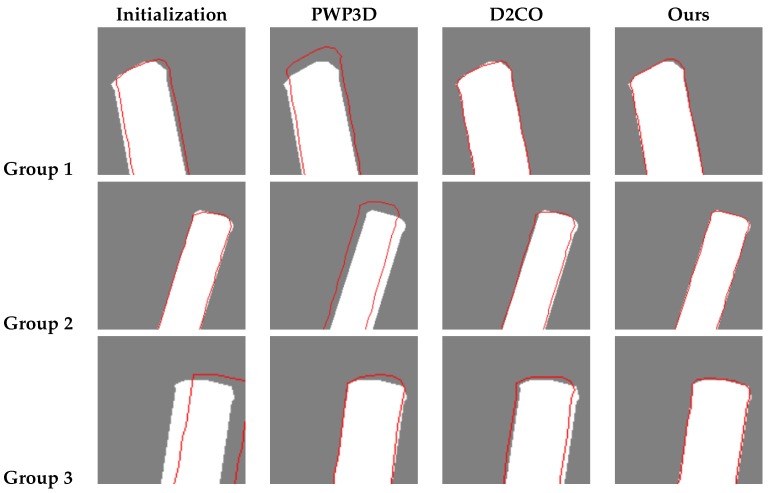
Examples from three groups of data (a single viewpoint for each) for which Pixel-Wise Posteriors for 3D tracking and segmentation (PWP3D) and direct directional chamfer optimization (D2CO) both yield unsatisfactory estimates (images are cropped because of space limitations). The white parts are antenna silhouettes in images, and the red curves are contours of antenna projections in estimated poses.

To ensure a fair comparison, we slightly modified the two existing methods. For PWP3D, we used the multi-view version and replaced the foreground/background probabilistic model with a deterministic one based on antenna silhouettes to eliminate errors in its segmentation step; for D2CO, we trivially extended it to obtain a multi-view version by accumulating the costs from each view, which is the strategy adopted in PWP3D. To initiate each method, we used our approximate poses, whose projections in each view show extensive overlap with the ground truth, as shown in the first column of Figure 12.

**Figure 13:**
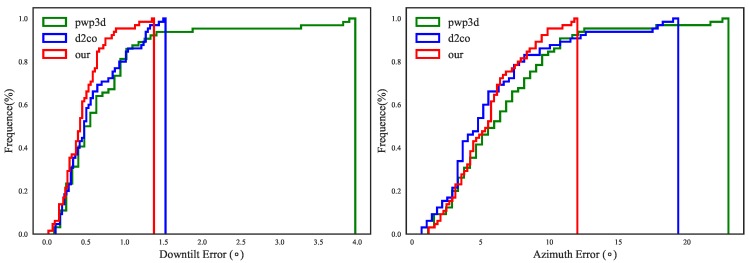
Error distributions for downtilt (left) and azimuth (right) estimation using the three methods.

In Figure 13, the distributions of the estimation errors are shown as cumulative histograms. We first note that all three methods yield satisfactory results on more than 90% of the data, which demonstrates the success of the initialization using our approximated poses. However, there are several exceptions in which PWP3D and D2CO fail to find the optimal poses, as shown toward the right end of the X axis ; the results for three of these cases are shown in Figure 12, together with the initial poses and the results of our method.

Table 3 presents the quantitative results for the three methods, where our method shows the lowest mean, standard and maximum errors.

### 6.3. Pose Estimation on Antennas

#### 6.3.1. Estimation with Camera-IMU Calibration

In this subsection, we evaluated the effects of the various camera-IMU calibration methods on the antenna downtilt and azimuth estimation accuracies using AntennaL. We performed the estimations using the pipeline proposed in this paper, in which we configured the camera-IMU calibration parameters offline using the results of the various calibration methods. For [37,50,51], we used the calibration results obtained from BoardF; for our method, we used the calibration results obtained from BoardL with *w* set to 0.1.

Table 4 compares the performances of all calibration methods in terms of the mean, standard and maximum errors for both downtilt and azimuth estimation. All four methods show improvements in downtilt estimation accuracy compared with an uncalibrated phone in terms of both the average and standard errors. Among them, the method proposed in [37] and our method yield results that are very close to the best result achieved using the method of [51], with mean/standard errors lower than 0.5/0.3. However, in terms of the azimuth accuracy, our method not only yields a reduction in the mean/standard error of up to 1.5, but also shows good control over the maximum error, making it the only method to achieve a lower azimuth error than the conventional maximum tolerance of 15 degrees, whereas the other methods do not show obvious improvements (compared with the default camera-IMU relation). These findings confirm the superior performance of our method in simultaneously improving both the downtilt and azimuth estimation accuracy.

#### 6.3.2. Extrinsic Camera Calibration from Pattern and SfM

We also evaluated the performance of our method when the camera is extrinsically calibrated using an SfM technique. We substituted the extrinsic camera parameters obtained from the checkerboards in Section 6.2.2 with results obtained from an SfM implementation in OpenMVG [55] while leaving all other details of the configurations unchanged.

**Figure 14:**
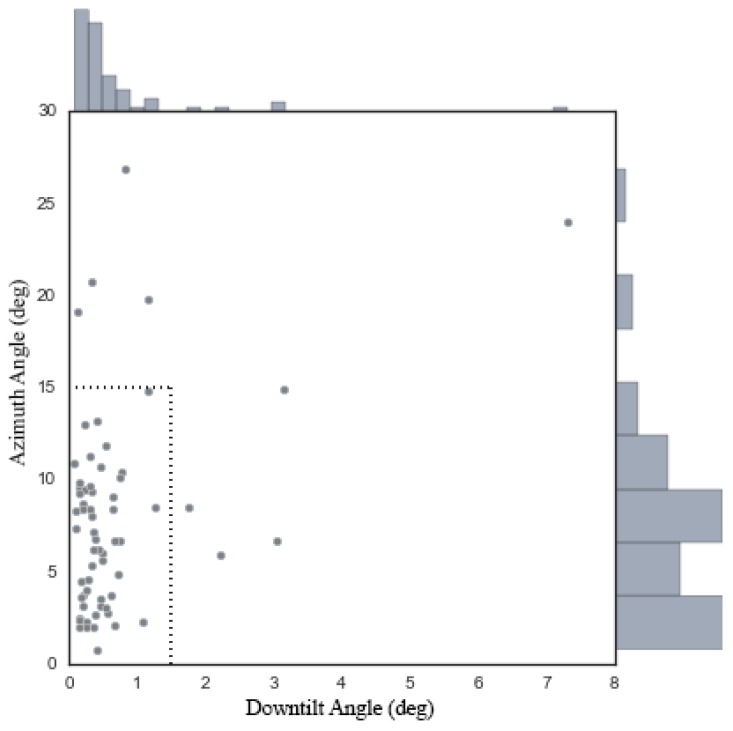
The estimation errors for each group of data from AntennaS when the camera was extrinsically calibrated using an SfM technique implemented in OpenMVG.

Figure 14 and Table 5 show the resulting estimation errors. Whereas nine out of 65 (or 13.8%) of the data cases failed to yield an estimate with a downtilt error of no more than 1.5 degrees and an azimuth error of no more than 15 degrees when the SfM technique was used, as seen from Figure 14, the majority of the results (at least 75%) show a precision comparable to that of the results obtained using a calibration pattern, as indicated by the first three quartiles reported in Table 5.

**Figure 15:**
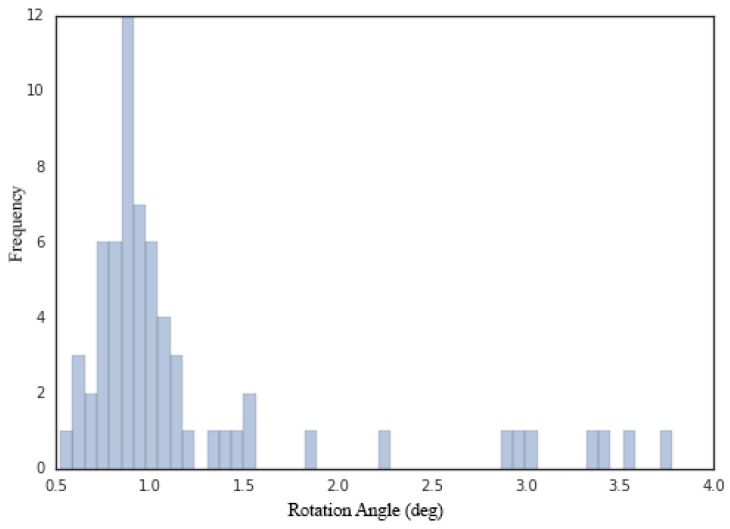
The rotation angles between the SfM calibrations and the pattern-based calibrations.

The few unsatisfactory estimates are strongly related to the accuracy of the SfM calibrations. To demonstrate this, we first measured the SfM accuracy against the calibration results obtained using the checkerboards by comparing the corresponding camera rotations relative to the first viewpoint for both calibration results for each data group, where the largest rotation angle was treated as the SfM accuracy. The histogram of the rotation angles is shown in Figure 15, in which seven of the nine data groups with the largest errors (larger than 1.5 degrees) also appear among the nine instances of unsatisfactory estimates, demonstrating the strong connection between the estimation error and the SfM accuracy.

**Figure 16:**
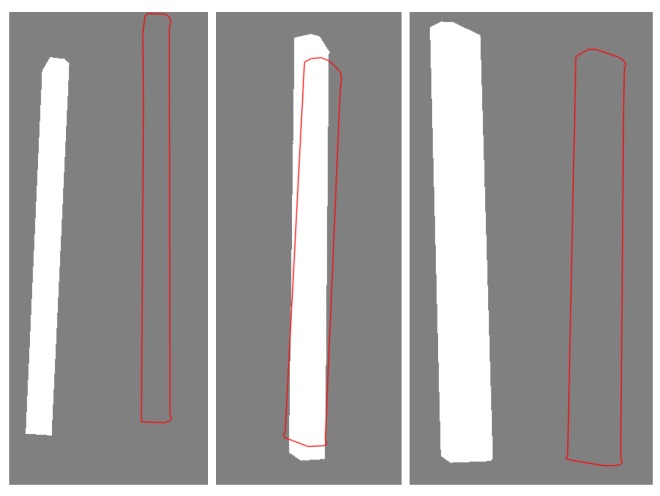
Three views of a situation in which the projection of the recovered 3D principal axis (on the red contour) is far from its real position and its center falls outside of the antenna silhouette.

Moreover, we find that the failures in the SfM experiment can be identified from the results of the coarse visual pose estimation stage: the projection of the center point of the recovered 3D principal axis falls outside of the antenna contour from at least one viewpoint, as demonstrated in Figure 16. This situation arises in all nine of the unsuccessful estimations and can be used to trigger an instruction to the end user to capture more images or to manually select matching features in the SfM procedure to overcome the problem.

#### 6.3.3. Performance on Working Antennas

In this subsection, we report an evaluation of our overall downtilt/azimuth estimation pipeline on working antennas to assess whether it satisfies the minimum accuracy requirements for industrial applications, i.e., to access whether the errors are lower than 1.5/15 degrees.

**Figure 17:**
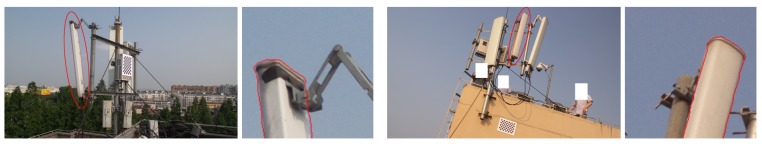
Two examples showing scenes with working antennas and the pose estimation results of our method.

The parameter configurations and evaluation method used are the same as those applied in the evaluations presented in Section 6.2.2. As demonstrated in Table 6, the largest errors on the antenna downtilt and azimuth angles estimated using our method are all below the tolerance values, demonstrating the applicability of our method in an industrial environment. Two typical visual pose estimation results are presented in Figure 17, where the red curves represent the projections of the 3D model based on the estimated poses.

## 7. Discussion and Conclusions

The focus of our study is the development of a novel non-contact solution for estimating antenna tilt and azimuth angles using a mobile phone as the measuring device. The two key points of our pipeline are the newly proposed camera-IMU calibration method for mobile phones and the coarse-to-fine visual pose estimation method.

The major difference between our camera-IMU calibration method and the state-of-the-art [37,50,51] is the inclusion of additional DoFs between the accelerometer frame and the magnetometer frame, which allows for decoupling of the accelerometer-related error and the magnetometer-related error and therefore leads to good performance on both tilt and azimuth estimation tasks simultaneously.

The crucial distinction between our visual pose estimation method and existing ones is the coarse-to-fine strategy we adopt. With this strategy, we avoid any manual pose initialization and more importantly are able to refine the approximate pose as a constrained optimization problem for higher accuracy compared with the state-of-the-art [32,35]. Besides, our method is based on multi-view contours instead of stable visual feature, which makes it very suitable for pose estimation of the textureless and simple-shaped antennas.

The major limitation of our work is the excessive computational resource consumption of the global optimization step of the pose refinement procedure. In the future, we will attempt to alleviate this problem by adding simple user interactions and/or developing more heuristic strategies for search space reduction.

We are also aware of the influence of hand shakes on accelerometer outputs if no tripod is used. According to our experience, a simple mean filter applied on the accelerometer data can effectively reduce the impact provided the shakes are slight; nevertheless, we intend to exploit methods used in image stabilization to fundamentally address the issue. Another related problem is the simple strategy for fusion of pose measurements from multiple viewpoints: though the present method of averaging works well in most cases, it may fail to generate the optimal results when outliers exist, as indicated by the relatively large error in the last row of Table 4. To overcome this problem, we have two working directions in the future: one is to adopt more powerful fusion methods, and the other is to integrate information from more sensors for an effective quality metric for pose measurements.

At last, we note that, aside from mobile telecommunications, our method can also be useful in areas such as the space field [64], indoor navigation [65], unmanned aerial vehicles [66], and so on.

## Figures and Tables

**Table 1:** Lower quartile (Q1), median (Q2) and the upper quartile (Q3) of error distribution of downtilt and azimuth estimation using the Broyden–Fletcher–Goldfarb–Shanno (BFGS) algorithm and constrained optimization by linear approximations (COBYLA) on AntennaL, together with the mean, standard devation (Std.) and maximum error are given for comparison.

	Downtilt		Azimuth	
	BFGS	COBYLA	BFGS	COBYLA
**Q1**	0.5	0.3	6.6	3.69
**Q2**	0.9	0.43	16.1	5.47
**Q3**	2.14	0.63	22.97	7.17
**Mean**	1.46	0.5	15.82	6.21
**Std.**	1.3	0.3	10.5	4
**Max**	5.24	1.23	42.17	22.46

**Table 2:** Comparisons of typical camera-IMU calibration methods in terms of downtilt and azimuth estimation. The values before and after the slash represent the mean error and standard error, respectively. The best results are shown in bold, and the next-to-best results are shown in bold italics.

Dataset	Type	Default	[37]	[50]	[51]	[44]	Ours^1^	Ours^2^
*hall*	**downtilt**	0.87/0.45	0.41/*0.28*	0.62/**0.25**	0.55/0.36	6.57/0.26	**0.39/*****0.28***	***0.40/0.28***
	**azimuth**	4.81/3.96	5.07/3.84	4.91/3.88	5.02/3.88	***4.37***/**2.21**	5.09/3.85	**3.87**/***2.90***
*balcony*	**downtilt**	0.52/0.27	0.36/**0.13**	0.74/0.27	**0.35**/*0.15*	3.89/2.23	***0.36***/**0.13**	***0.36***/**0.13**
	**azimuth**	4.39/4.42	5.60/4.82	***4.24***/**2.35**	5.29/4.71	6.45/5.45	5.30/4.53	**3.52**/***2.41***
*BoardF*	**downtilt**	0.86/0.44	**0.41**/***0.28***	0.57/**0.25**	0.55/0.37	4.48/2.34	**0.41/*****0.28***	***0.42/0.28***
	**azimuth**	5.51/4.44	5.52/4.50	5.53/***4.43***	***5.50***/4.53	5.93/4.46	5.53/4.54	**3.51/2.18**

**In Ours^1^:** w=0; **in Ours^2^:** w=0.1.

**Table 3:** Estimation errors achieved using PWP3D, D2CO and our method.

	Downtilt			Azimuth		
	PWP3D	D2CO	Ours	PWP3D	D2CO	Ours
**Mean**	0.93	0.6	**0.47**	6.83	5.98	**5.6**
**Std.**	1.37	0.38	**0.27**	4.35	4.23	**2.62**
**Max**	10.08	1.53	**1.38**	23.01	19.36	**12.02**

**Table 4:** Comparison with state-of-the-art camera-IMU calibration methods in terms of antenna downtilt and azimuth angles.

	Downtilt					Azimuth				
Method	Default	[37]	[50]	[51]	Ours	Default	[37]	[50]	[51]	Ours
**Mean**	0.87	0.49	0.76	**0.45**	0.47	7.14	7.19	7.26	7.18	**5.60**
**Std.**	0.39	0.29	0.36	**0.26**	0.27	4.20	4.14	4.17	4.13	**2.62**
**Max**	2.08	1.41	2.12	**1.32**	1.38	17.17	17.93	18.23	17.84	**12.02**

**Table 5:** Comparison of estimation errors from cameras extrinsically calibrated using an SfM technique and with a calibration pattern. The pattern-based results are duplicated from Table 3.

	Downtilt		Azimuth	
	SfM	Pattern	SfM	Pattern
**Min**	0.08	0.02	0.79	1.20
**Q1**	0.22	0.27	3.63	3.70
**Q2**	0.36	0.43	6.74	5.59
**Q3**	0.64	0.61	9.60	6.97
**Mean**	0.66	0.47	7.84	5.60
**Std.**	1.03	0.27	5.45	2.62
**Max**	7.32	1.38	26.90	12.02

**Table 6:** Downtilt and azimuth estimation results for our method when applied to working antennas.

	Mean	Std.	Max
**Downtilt**	0.61	0.31	1.13
**Azimuth**	8.7	2.25	12.2
